# Inhaled tobramycin for the treatment of nosocomial pneumonia in sepsis

**DOI:** 10.1186/cc13550

**Published:** 2014-03-17

**Authors:** A Kuzovlev, V Moroz, A Goloubev

**Affiliations:** 1 V.A. Negovsky Scientific Research Insitute of General Reanimatology RAMS, Moscow, Russia

## Introduction

Nosocomial pneumonia (NP) is frequently caused by multiresistant Gram-negative bacteria. The inhaled antibiotics provide us with a new treatment modality for NP in sepsis. The aim of this study was to estimate the efficacy of inhaled tobramycin (IT) as an adjunct to systemic antibiotics in the treatment of NP in sepsis.

## Methods

Fifty ICU ventilated septic patients with NP were enrolled in the study (all male, 55.3 ± 7.3 years old; primary reason for ICU stay - intraabdominal infections (78%), mediastinitis (13%), other (9%)). Diagnosis of NP was made according to the standard clinical and CPIS criteria. Associations of multiresistant Gram-negative bacteria were detected in bronchoalveolar lavage (BAL) of all patients. Seventy- two percent of bacteria were sensitive to tobramycin. Patients were randomized into two groups: IT (group 1, *n *= 25), addition of IT to systemic antibiotics (carbapenems, aminoglycosides, protected penicillins); and no IT (group 2, *n *= 25), shift of systemic antibiotics according to sensitivity. Groups were comparable in APACHE II and CPIS scores. IT (Bramitob) was administered 300 mg twice daily via nebulizer. The data were statistically analyzed by STATISTICA 7.0 (M, σ, Newman- Keuls test; *P *< 0.05).

## Results

Administration of IT as an adjunct to systemic antibiotics was associated with a decrease of systemic inflammation and acute respiratory insufficiency signs 2.3 ± 1.2 days after the treatment onset (vs. 6.3 ± 1.5 days in group 2, *P *= 0.03). The decrease of microbial titer to 10^3 ^to 10^4 ^CFU/ml was detected in both groups by days 5 to 7, but it was reliable in 80% of the patients of group 1 (*P *< 0.02). It is noteworthy that 21% of group 1 patients were *in vitro *resistant to tobramycin, but it was clinically effective, probably due to a local superconcentration. Treatment with IT was associated with an increase of sensitivity of microbes to antibiotics they were prior resistant to (32% of patients). This is probably due to IT effects on biolayers. De-escalation of antibiotic therapy was possible in group 1 by day 5 in 42% of patients. The treatment with IT made it possible to wean 40% of patients by day 5.3 ± 1.8 after treatment cessation (vs. 35% and 11.2 ± 1.3 days in group 2, *P *= 0.02). Hearing loss and tinnitus was detected only in three patients of group 1. There were no cases of bronchospasm. The mortality was 12% (*n *= 3) in group 1 and 16% (*n *= 4) in group 2 (*P *> 0.05), and was not related to a progression of NP [1].

## Conclusion

Administration of IT as an adjunct to systemic antibiotics is efficient in treatment of NP caused by multiresistant Gram-negative bacteria in sepsis.
